# What we need is person-centred care

**DOI:** 10.1007/s40037-017-0334-4

**Published:** 2017-02-20

**Authors:** Heather Stuart

**Affiliations:** Departments of Public Health Sciences, Psychiatry, and the School of Rehabilitation Therapy, Queen’s University, Kingston, Ontario Canada

Sukhera and colleagues [1] have tackled a thorny issue. Despite good intentions, health care providers are among the groups most often identified by people with a mental illness as stigmatizing toward them. Recurrent themes in the literature that fall under the rubric of stigma are that patients feel as if they were being patronized, humiliated, treated like children, excluded from treatment decisions, being assumed to lack capacity to be responsible for their own lives and treatment decisions. Other problems include not being given sufficient information about their illness and treatment options, prognostic negativism, and at times, the unspoken threat of coercive treatment [2]. As this paper points out, this has as much to do with accumulated organizational practices as it does with individual biases and personal prejudices.

We have known for some time that prejudices are resistant to change, particularly prejudices that are rooted in fear of unpredictability and violence. Yet, many anti-stigma programs draw on ‘off-the-shelf’ educational solutions without ever considering the underlying theory that might explain why a given intervention might be effective. Thus, programs abound that try to raise awareness or correct stereotypic beliefs. However, there is no supporting evidence from social psychology to suggest that prejudices are responsive to such information. People will selectively attend to information that supports their prejudices and negative habits and actively discount information that contradicts them [3]. As vividly illustrated in the case of health providers, good mental health literacy can coexist with high levels of social intolerance. By changing one, you do not necessarily change the other [4]. Pescosolido et al. [5] examined changes in knowledge about the causes of mental illness and public stereotypes between 1996 and 2006 in the United States. In 2006, a greater proportion of the public embraced ‘professional’ neurobiological explanations for mental illnesses such as schizophrenia, depression, or alcohol dependence. Public endorsement for medical treatments also increased. However, there was no corresponding decrease in public levels of intolerance, which remained high. The majority of respondents continued to express a desire for social distance. Of interest was that neurobiological explanations were either unrelated to intolerance or increased the odds of a stigmatizing reaction.

So, if more information won’t work, then what is the solution? Sukhera et al. make the important point that educational interventions must do more than simply educate. They must alter the very nature of the patient-provider relationship as well as the socio-cultural context in which these occur.

The Opening Minds anti-stigma initiative of the Mental Health Commission of Canada [6] has identified six key ingredients in anti-stigma programs that promote more humanizing, patient-centred relationships. The most successful programs demonstrated the potential for recovery from mental illnesses using real life examples and interactions with people who demonstrate that recovery happens. The resulting transformative learning experience replaced negative expectations that individuals with a mental illness are ‘unfixable’, with positive expectations of recovery. In this context, recovery is understood as living a satisfying life within the constraints of a mental illness.

The development of competencies needed to undertake recovery oriented care has been used in mental health systems to assist in changing the way providers think about and work with their clients [7]. An understanding about recovery can be promoted through structured educational initiatives (preferably involving people with lived experience of a mental illness), conferences, newsletters, and, perhaps most importantly, changes to academic curricula. Indeed, education about recovery and recovery oriented care can be targeted broadly to service providers in health and social services agencies, people who have experienced a mental illness, their family members, and policy makers [8].

In their examination of recovery competencies for inpatient care, Chen and colleagues [7] also identified the physical environment as undermining recovery oriented care where routines and interactions are experienced as dehumanizing, discouraging, and disempowering. As Sukhera et al. point out; the physical environment is also a major challenge for emergency personnel. In busy emergency departments, mental health clients may be triaged to the ‘bottom of the list’ and end up waiting for an excessive amount of time to see a provider. A typical emergency room environment would be experienced as over stimulating and frightening for someone with a serious mental illness, and may fuel psychosis and feelings of agitation. Psychiatric labels may also get in the way of appropriate physical care as clients are triaged as ‘psychiatric’ regardless of their physical needs and presenting complaint [9]. Finally, it is important to also recognize that emergency departments function as a key portal of entry into a system of care that is perceived by many people with a serious mental illness as coercive, as initial decisions about involuntary commitment are typically made in emergency departments. Coercion has been identified as one of the most detrimental barriers to recovery and a key reason why people delay or avoid seeking care [10].

Outside of the mental health field, the notion of ‘person-centred care’ has gained considerable currency as a model of care and it is beginning to exert an influence on policy and practice [11]. Person-centred care is depicted as care that values people, respects people as individuals, and organizes care to meet their needs. To provide person-centred care one must be able to value the totality of the individual. This would require health professionals to move beyond roles and concepts that are cure-based with a focus on scientific and technical competence, to a holistic value-based approach with a focus on interpersonal competencies. Person-centred care is also based on the idea that the patient is not a passive recipient of care but an active partner. Providing person-centred care in an emergency department would require providers to change their traditional role of addressing the urgent physical needs of clients, to one that embraces mental, emotional, spiritual, and social aspects of patient care.

The confluence of the principles of the recovery paradigm in the mental health system, and person-centred care in the broader health care system suggests that it is now time to create a health care environment that ensures that patients and their family members receive the care that meets their needs, delivered in ways that are affirming and recovery-oriented rather than disempowering and stigmatizing.
